# Guinea pigs raised as livestock are incidental host of *Toxoplasma gondii* and Influenza A in Ecuador

**DOI:** 10.3389/fvets.2025.1657510

**Published:** 2025-09-29

**Authors:** Mauricio Xavier Salas-Rueda, Patricia Peralta-Ortiz, Jairo Guama-Tipas, Katherine Natalia Chávez Toledo, Mónica Espadero, Pedro Webster, Juan Masache-Masache, Karla Monica Illescas Sigcha, Fabiola Estefania Yungazaca Jaramillo, Angel Sebastian Rodríguez-Pazmiño, Fabricio Arcos Alcivar, Solon Alberto Orlando, Miguel Ángel Garcia-Bereguiain

**Affiliations:** ^1^Universidad Politécnica Salesiana, Cuenca, Ecuador; ^2^Clínica Veterinaria El Gran Pastor, Cuenca, Ecuador; ^3^Clínica Veterinaria Wasi Vet, Ibarra, Ecuador; ^4^Universidad Católica Santiago de Guayaquil, Guayaquil, Ecuador; ^5^Gobierno Autónomo Descentralizado de Sayausi, Cuenca, Ecuador; ^6^One Health Research Group, Universidad de Las Américas, Quito, Ecuador; ^7^Universidad Ecotec, Guayaquil, Ecuador; ^8^Instituto Nacional de Salud Pública e Investigación, Guayaquil, Ecuador; ^9^Universidad Espíritu Santo, Guayaquil, Ecuador

**Keywords:** zoonosis, guinea pigs, *Toxoplasma gondii*, Influenza A, Ecuador

## Abstract

The guinea pig (*Cavia porcellus*) is commonly used as a laboratory model or kept as a pet in many Western countries; however, in Andean countries like Ecuador, it is raised as livestock. Despite its importance to rural local economies, specific management guidelines for guinea pig farming have not been enforced by animal or public health authorities. Several reports indicate that guinea pigs raised as livestock serve as incidental host for respiratory and enteric pathogens, including *Toxoplasma gondii*. This study analysed the seroprevalence of antibodies against several pathogens relevant to public health and animal production in Ecuador: Influenza A, *Brucella* spp., *Coxiella burnetii*, *Toxoplasma gondii*, and *Neospora caninum.* Blood samples from 240 guinea pigs were collected in the cantons of Cuenca, Paute, and Gualaceo, in the Azuay province of Ecuador. Seropositive animals were detected for two pathogens—Influenza A and *T. gondii*—with prevalence rates of 1.67% (95% CI: 0.46–4.21) and 16.25% (95% CI: 11.82–21.54), respectively. There were not seropositive animals for *Brucella* spp.*, Coxiella burnetii* and *Neospora caninum.* These results underscore the potential role of guinea pigs as incidental host for Influenza A and support their inclusion in surveillance programs for panzootic flu outbreaks. Additionally, guinea pigs may play a significant role in the epidemiology of toxoplasmosis in the Andean regions of Ecuador, Peru, and Colombia, where similar findings have been reported.

## Introduction

Although the guinea pig (*Cavia porcellus*) is commonly used as a laboratory model or kept as a pet in many Western countries (1), it is raised as livestock in Andean countries of South America, including Colombia, Ecuador, Peru, and Bolivia (2). Its meat is valued for its low fat and high protein content (3), but guinea pigs are still bred using traditional methods, often raised inside rural homes in groups of up to 50 animals per household. Additionally, more industrialized farms housing thousands of animals also exist. In Ecuador alone, at least 700,000 families are involved in guinea pig farming, with an estimated annual production of 47 million animals (4, 5). However, specific management guidelines for guinea pig farming have not been implemented by animal or public health authorities to ensure food safety and quality (6).

Although there is extensive literature on the use of guinea pigs as models for infectious disease research and zoonotic transmission from pet guinea pigs, there is limited information on guinea pigs raised as livestock (6). Nevertheless, several studies have reported the presence of respiratory pathogens such as yeasts, Influenza virus, Methicillin-Resistant *Staphylococcus aureus* (MRSA), and *Streptococcus pneumoniae* in livestock guinea pigs (7–13). Guinea pigs also act as zoonotic reservoirs for enteric bacterial pathogens such as *Campylobacter jejuni* (14), and parasites including *Blastocystis, Entamoeba*, and *Cryptosporidium* (15). Furthermore, two recent studies from Colombia and Peru have described, for the first time, the role of guinea pigs as reservoirs for *Toxoplasma gondii*, the parasite responsible for toxoplasmosis (16, 17). *T. gondii* has a life cycle including a sexual cycle within a feline definitive host and an asexual cycle with a wide range of intermediate avian and mammal hosts (16, 17). Oocyst are produced within the intestines of felines and shed with their feces into the environment; when ingested by intermediate hosts, oocysts develop into infective tachyzoites that penetrate host tissue to form cysts of slow growing bradyzoites (16, 17). Infection of definitive or intermediate host may also happened through the ingestion of tissue cysts of infected animals (16, 17). These findings are particularly concerning for food safety, given the high prevalence rates (over 20%) detected in organs like muscle (16, 17). Other zoonotic diseases like bartonelosis (18), leishmaniases (19) and brucelosis (20) have also been associated to guinea pigs.

Other zoonotic diseases, such as brucellosis and Q-fever, have been reported in Ecuador in association with cattle, wildlife, and free-roaming dogs (21–26). Brucellosis, caused by bacteria of the genus *Brucella*, and Q-fever, caused by *Coxiella burnetii*, are both panzootic pathogens capable of infecting multiple mammalian species including wild fauna (26, 27). The use of guinea pigs as models to study these diseases underscores their susceptibility to infection (28, 29). Additionally, the parasite *Neospora caninum* is also highly prevalent in cattle, free-roaming dogs and wild mammals (21, 30, 31). Although not zoonotic, it causes reproductive problems in livestock (21). To the best of our knowledge, there is no existing information on the presence of the aforementioned pathogens in guinea pigs raised as livestock.

In this context, the aim of this study is to characterize the seroprevalence of several pathogens relevant to animal production and public health in guinea pigs raised as livestock in Ecuador. These diseases include Influenza A, brucellosis, Q-fever, toxoplasmosis and neosporosis, relevant pathogens to public and animal health that support the idea of improving the One Health perspective within guinea pig farming.

## Materials and methods

### Study design and setting

This was a cross-sectional study including guinea pigs from the Azuay province. This province, located in the Andean region of Ecuador at 2,500 meters above sea level, is one of the country’s main producers of guinea pig meat (32). Samples were collected from guinea pig slaughterhouses in three cantons of Azuay province based on accessibility: Paute (89 samples), Gualaceo (12 samples), and Cuenca (139 samples). Sample collection was conducted throughout 2022.

### Animal selection

Blood samples were obtained from 240 healthy guinea pigs. Animals were selected based on convenience sampling at slaughterhouses, depending on the availability of these facilities to permit sample collection. There were no exclusion criteria, and any animal slaughtered while we were present was included in the study. Although convenience sampling method have the limitation of potential selection bias, it was the only possible approach to carry out this study as guinea pig breeders were not willing to allow blood sample collection out of the slaughterhouses.

### Sample collection

Two milliliters of blood were collected from the jugular vein using red-top tubes containing serum clot activators. Veterinary staff carrying protective gear and sterile material was used for sample collection to guarantee an aseptic blood extraction. The samples were stored at 4 °C (ice bucket with termometer to control temperature) within 1 min after collection and transported to the laboratory within 2 h after collection. After clotting, 1 to 1.5 mL of serum was separated and transferred into 2 mL Eppendorf tubes. Serum samples were stored at −20 °C until analysis within 5 h after collection.

### Laboratory analysis

Commercial indirect ELISA kits, “ID Screen® Brucellosis Serum Indirect” (lot number: BRUS-MS-5P J67; expiration date: 03/2024), “ID Screen® Q Fever Indirect Multi-species” (lot number: FQS-MS-5P K69; expiration date: 11/2024), “ID Screen® Influenza A Antibody Competition Multi-species” (lot number: INFS-MS-5P G44; expiration date: 02/2024), “ID Screen® Toxoplasmosis Indirect Multi-species” (lot number: TOXOS-MS-2P K35; expiration date: 06/2024) and” ID Screen ® *Neospora caninum* Competition Multi-species”(lot number: NEOS-MS-5P K47; expiration date: 10/2024) (IDVet, France), were used to detect antibodies against *Brucella* spp. (*B. abortus, B. melitensis*, or *B. suis*), *Coxiella burnetii*, Influenza A, *Toxoplasma gondii*, and *Neospora caninum*, respectively (See Supplementary Table 1). The procedures were carried out in accordance with the manufacturer’s instructions.

The optical density cut-off values for determining positive and negative results were based on the manufacturer’s instructions. The S/P ratio, calculated as the sample optical density relative to the positive control provided with the kit, was used to determine results based on the following cut-off values: (1) for Brucellosis: S/P % ≤ 110% negative, 110% < S/P % < 120% inconclusive, S/P % ≥ 120% positive; (2) for Q-fever: S/P % ≤ 40% negative; 40% < S/P % < 50% inconclusive; 50% < S/P positive; (3) for Influenza A: S/P % ≤ 45% negative, 45% < S/P % < 50% inconclusive, S/P % ≥ 50% positive; (4) for Toxoplasmosis: S/P % < 40% negative, 40% < S/P % < 50% inconclusive, S/P % > 50% positive; for *Neospora caninum*: S/P % ≤ 40% negative, 40% < S/P % < 50% inconclusive, S/P % ≥ 50% positive. All samples were tested in duplicate. In cases where both replicates were “inconclusive” (as per manufacturer’s manual definition), a third replicate was performed. If the result remained “inconclusive,” it was recorded as negative. As far as we could not confirm a sample as positive and without any other diagnosis tool available, we preferred to consider positive samples only those ones with conclusive results. The performance metrics of the ELISA kits were inferred from the validated species mentioned above. Moreover, to avoid samples cross-contamination, a reference negative serum (provided with the kit) was always processed within every set of guinea pig samples.

According to the manufacturer, the ELISA kit for Brucella spp. antibodies has a sensitivity of 100% (95% CI: 89.57–100%) and a specificity of 99.74% (95% CI: 99.24–99.91%); the ELISA kit for *C. burnetii* has a sensitivity of 100% (C.I. 95%: 89.28–100%) and a specificity of 100% (C. I. 95%: 97.75–100%); the ELISA kit for Influenza A has a sensitivity of 97.30% (C. I. 95%: 86.18–99.52%) and specificity of 100% (C. I. 95%: 99.36–100%); the ELISA kit for *T. gondii* has a sensitivity of 98.36% (C. I. 95%: 95.30–99.40%) and a specificity of 99.42% (C. I. 95%: 98.50–99.70%); the ELISA kit for *N. caninum* has a sensitivity of 100% (C. I. 95%: 98.10–100%) and a specificity of 100% (C. I. 95%: 97.70–100%) (See Supplementary Table 1).

All these IDVet kits have been validated in ruminants (cattle, sheep, goats), pigs and dogs. According to the supplier, the kits employ conjugates that detect anti-mammalian antibodies. So, we have used these kits in guinea pigs based on cross-reactivity of anti-mammalian conjugates.

### Statistical analysis

The data were processed and analysed using EpiInfo version 7.2.5.0. Prevalence percentages along with 95% confidence intervals (Wilson score method as per software default settings) were calculated. Chi-square test was used to compare prevalence among cantons. A *p*-value of < 0.05 was considered statistically significant. For missing data in each ELISA kit test, the sample was eliminated from further analysis. As we have stated above, inconclusive results were considered negative for statistical analysis.

### Ethical considerations

According to national regulations in Ecuador, no IRB approval is needed for surveillance and diagnosis of diseases in domestic animals. The animal handling was carried by certified veterinarians following standard procedures for animal welfare.

## Results

**Figure 1:**
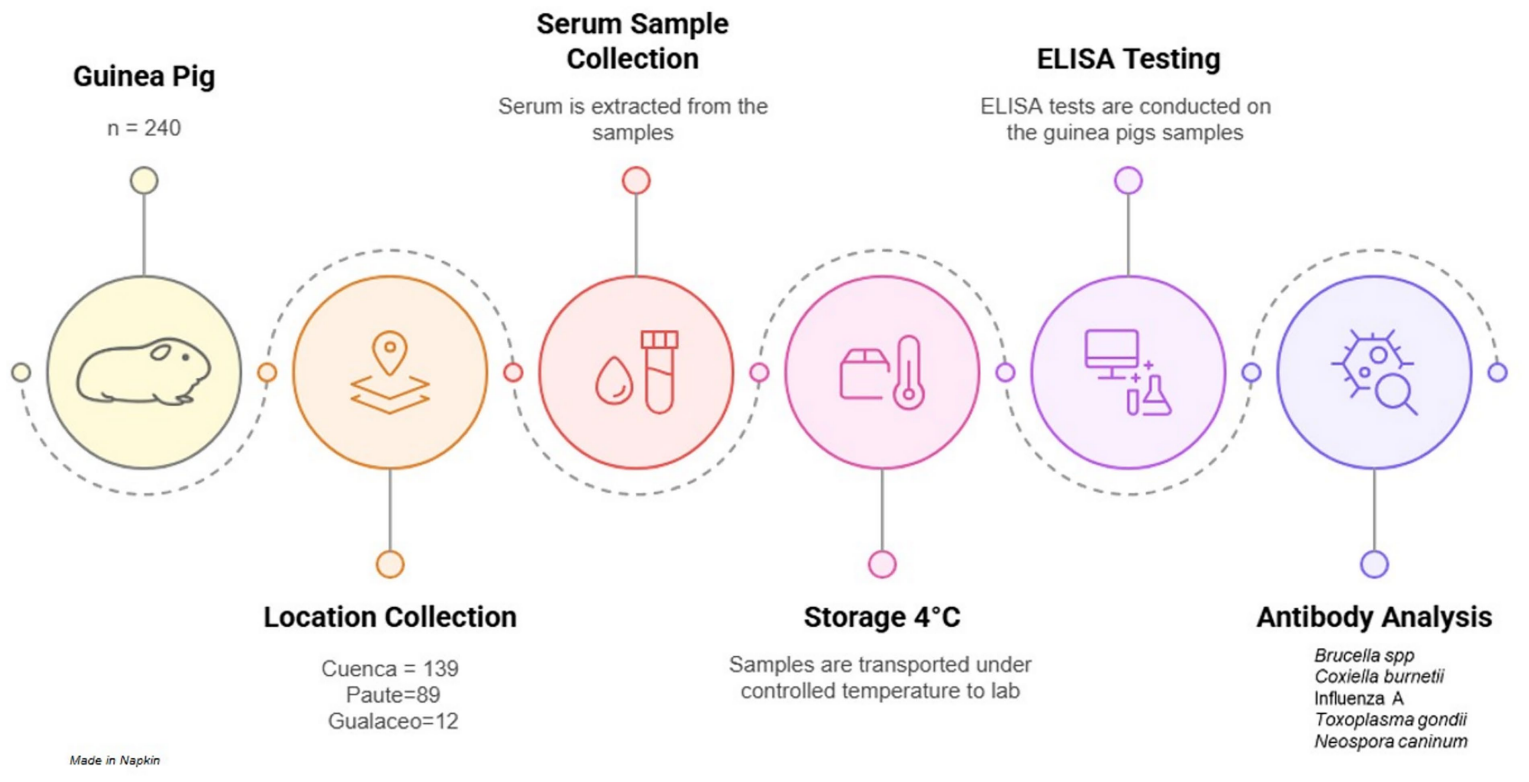
Flow diagram from sample collection and processing in our study.

A total of 240 guinea pigs were included in the study, distributed across three cantons in Azuay province: 139 from Cuenca, 89 from Paute, and 12 from Gualaceo. The seroprevalence results for antibodies against Influenza A, *Brucella* spp., *Coxiella burnetii*, *Toxoplasma gondii*, and *Neospora caninum* in each canton are presented in Table 1. The study’s flow diagram is shown in Figure 1.

**Table 1:** Seroprevalence of antibodies against the 5 pathogens causing the diseases included in the study for guinea pigs.

Location	*n*	*Neopora caninum*	*Brucella* spp.	*Coxiella burnetii*	*Toxoplasma gondii*	Influenza A
Gualaceo	1	0.0% (0/12)	0.0% (0/12)	0.0% (0/12)	25.00% (3/12)	0.0% (0/12)
	2	–	–	–	IC (5.49–57.19%)	–
Paute	8	0.0% (0/89)	0.0% (0/89)	0.0% (0/89)	14.61% (13/89)	0.0% (0/89)
	9	–	–	–	IC (8.01–23.68%)	–
Cuenca	1	0.0%	0.0%	0.0%	16.55 (23/139)	2.88% (4/139)
	3	(0/139)	(0/139)	(0/139)	IC (10.79–23.79%)	IC (0.79–7.20%)
	9	–	–	–		
Total	240	0.0%	0.0%	0.0%	16.25% (39/240)	1.67% (4/240)
	4	(0/240)	(0/240)	(0/240)	IC (11.82–21.54%)	IC (0.46–4.21%)
	0	–	–	–		

No seropositive animals were detected for three of the pathogens: *Brucella* spp., *Coxiella burnetii,* and *Neospora caninum*. No inconclusive samples were found.

Four guinea pigs tested seropositive for antibodies against Influenza A. The overall seroprevalence was 1.67% (95% CI: 0.46–4.21). All four seropositive animals were from the Cuenca canton. The prevalence in Cuenca was 2.88% (4/139) (95% CI: 0.79–7.20). No inconclusive samples were found.

Thirty-nine guinea pigs tested seropositive for antibodies against *T. gondii*. The overall seroprevalence was 16.25% (95% CI: 11.82–21.54). The 39 seropositive animals were distributed among all three cantons: Cuenca (23/139, 16.55%; 95% CI: 10.79–23.79), Paute (13/89, 14.61%; 95% CI: 8.01–23.68), and Gualaceo (3/12, 25.00%; 95% CI: 5.49–57.19). The differences in *T. gondii* antibody prevalence among the three cantons were not statistically significant (*p* = 0.65). Two inconclusive samples were found, and as it was detailed in the method, these samples were considered negative.

## Discussion

Guinea pigs have been shown to be incidental hosts for zoonotic transmission of respiratory pathogens of public health concern, including yeasts, Influenza virus, Methicillin-Resistant *Staphylococcus aureus* (MRSA), and *Streptococcus pneumoniae* (7–13). A pioneering study conducted in Ecuador in 2012 reported a high prevalence of antibodies against Influenza A and B in guinea pigs from markets in Manabí Province, Guayaquil, and Cuenca (8).

Our study confirms previous findings on the circulation of Influenza A in guinea pigs in Ecuador. This fact has implications for the ongoing panzootic of highly pathogenic avian Influenza A H5 in the Americas (33–35). The current panzootic has caused outbreaks not only in wild birds and poultry but also in several mammalian species, including dairy cows (36). By one hand, there is circulation of Influenza A and B in guinea pigs in Ecuador. On the other hand, intensive small mammal farming (minks) and explosive H5 influenza outbreaks have been reported in Europe (37). In this context, sentinel surveillance for avian and swine influenza should extend beyond poultry and pigs to include guinea pig.

Two recent studies from Colombia and Peru were the first to report the role of guinea pigs as reservoirs for *Toxoplasma gondii* (16, 17). These findings are particularly concerning due to the high *T. gondii* prevalence—23.3% in Cuzco, Peru, and 27.5% in Nariño, Colombia (16, 17). Moreover, these prevalence rates were based on PCR detection of *T. gondii* DNA, confirming active infection (16, 17). Additionally, *T. gondii* DNA was detected in multiple tissues, particularly in the brain, as well as the heart and muscle (16, 17). Our study demonstrated the circulation of *T. gondii* in Ecuadorian guinea pigs at a high prevalence (over 16%), corroborating previous findings from Peru and Colombia (16, 17). Guinea pigs are traditionally cooked and consumed whole, including the head, where the parasite was found at higher prevalence (16, 17). Our study, along with those from Peru and Colombia, underscores the need for public and animal health authorities to recognize guinea pigs as incidental host for zoonotic transmission of toxoplasmosis in the Andean region. From a food safety perspective, thorough cooking of guinea pigs should be recommended to prevent infection.

It is important to emphasize that *Brucella* spp., *Coxiella burnetii*, or *Neospora caninum* was not found in guinea pigs in our study. However, these three pathogens have been reported at high prevalence in cattle, wildlife, and free-roaming dogs in Ecuador and elsewhere (21–31). Further studies are needed to confirm the absence of this pathogens in guinea pigs considering their presence in livestock in Ecuador. Nevertheless, restricting access of all domestic animals, including pets, should be established as mandatory policy in guinea pig farms. Such measures would help prevent the spill over of zoonotic diseases like Q-fever and brucellosis to guinea pigs.

Our study has some limitations that we would like to acknowledge. Sampling was done at convenience and location was selected based on accessibility granted for blood collection. These facts may introduce sample bias. Also, the sample size for Gualaceo canton was only 12 animals, and that may also introduce sample bias. As sample collection was done at slaughterhouses, no risks factor analysis was possible as we could not identify the farm of origin of each animal. So far, further studies with larger population of guinea pigs are recommended. Moreover, the diagnosis was based on serology and molecular or microbiological diagnostics is recommended to confirm active infections in further studies.

In conclusion, guinea pigs raised as livestock in Ecuador serve as incidental host for the zoonotic transmission of Influenza A and *T. gondii*. The growing body of literature on guinea pig health and production highlights the need for increased awareness regarding their role as zoonotic reservoirs. However, the number of studies remains limited, and most are local and descriptive, lacking analysis of potential risk factors associated with pathogen presence. Future research should include larger sample sizes across multiple provinces in Ecuador and incorporate risk factor analyses related to guinea pig farming—such as proximity to other livestock, presence of domestic animals within farms, and farmers’ use of protective equipment. Such studies are essential to better understand the public health risks associated with guinea pig farming and to develop evidence-based guidelines for improving guinea pig health and production within a One Health framework.

## Data Availability

The original contributions presented in the study are included in the article/Supplementary material, further inquiries can be directed to the corresponding author.
