# Supplementary material for: Guinea pigs raised as livestock are incidental host of Toxoplasma gondii and Influenza A in Ecuador
Source: Front Vet Sci. 2025 Sep 29;12:1657510. doi: 10.3389/fvets.2025.1657510 (PMC12515611; doi:10.3389/fvets.2025.1657510)
Supplement: Supplementary file 1 [file Data_Sheet_1.pdf]

Supplementary Table 1. ELISA Kits used in the study (data provided from IDVet company manuals).

Pathogen	ELISA kit	lot number	expiration date	Sensitivity (CI 95%)	Specificity (IC 95%)
Brucella spp	ID Screen® Brucellosis Serum Indirect	BRUS-MS-5P J67	03/2024	100% (89.57–100%)	99.74% (99.24–99.91%)
C. burnetii	ID Screen® Q Fever Indirect Multi-species	FQS-MS-5P K69	11/2024	100% (89.28–100%)	100% (97.75–100%)
Influenza A	ID Screen® Influenza A Antibody Competition Multi-species	INFS-MS-5P G44	02/2024	97.30% (86.18–99.52%)	100% (99.36–100%)
T. gondii	ID Screen® Toxoplasmosis Indirect Multi-species	TOXOS-MS-2P K35	06/2024	98.36% (95.30–99.40%)	99.42% (98.50–99.70%)
N. caninum	ID Screen ® *Neospora caninum* Competition Multi-species	NEOS-MS-5P K47	10/2024	100% (98.10–100%)	100% (97.70–100%).
